# Hickam’s Dictum Incarnate: A Case of Simultaneous Left-Sided Urolithiasis and Ruptured Iliac Artery Aneurysm

**DOI:** 10.24908/pocus.v7i1.15020

**Published:** 2022-04-21

**Authors:** Melissa Bouwsema, Colin Bell

**Affiliations:** 1 Department of Emergency Medicine, Queen's University Kingston, ON Canada; 2 Department of Emergency Medicine, University of Calgary Cumming School of Medicine Calgary, AB Canada

**Keywords:** urolithiasis, nephrolithiasis, iliac artery aneurysm, abdominal aortic aneurysm, ultrasound, point of care ultrasound

## Abstract

A 51-year-old man with a history of nephrolithiasis presented to the Emergency Department after a sudden onset of left-sided groin pain and syncope. At presentation, he described his pain as similar to prior renal colic episodes. At his initial assessment, point of care ultrasound (POCUS) was used, which revealed findings consistent with obstructive renal stones, as well as a substantially enlarged left iliac artery. Computed tomography (CT) imaging confirmed the comorbid diagnoses of left-sided urolithiasis and a ruptured isolated left iliac artery aneurysm. POCUS facilitated expedited definitive imaging and operative management. This case highlights the importance of performing related POCUS studies in reducing anchoring and premature closure bias.

## Case File

A 51-year-old man with a previous history of renal stones and gout presented to the emergency department after sudden onset severe 10/10 left-sided groin pain accompanied by a syncopal episode. His triage vitals were BP 126/87, HR 92, RR 20, SpO_2_ 97% on room air, T36.5°C. On assessment, the pain had improved, and the patient was complaining of 2/10 left groin pain, stating the discomfort was similar to previous renal colic episodes. 

**Figure 1:**
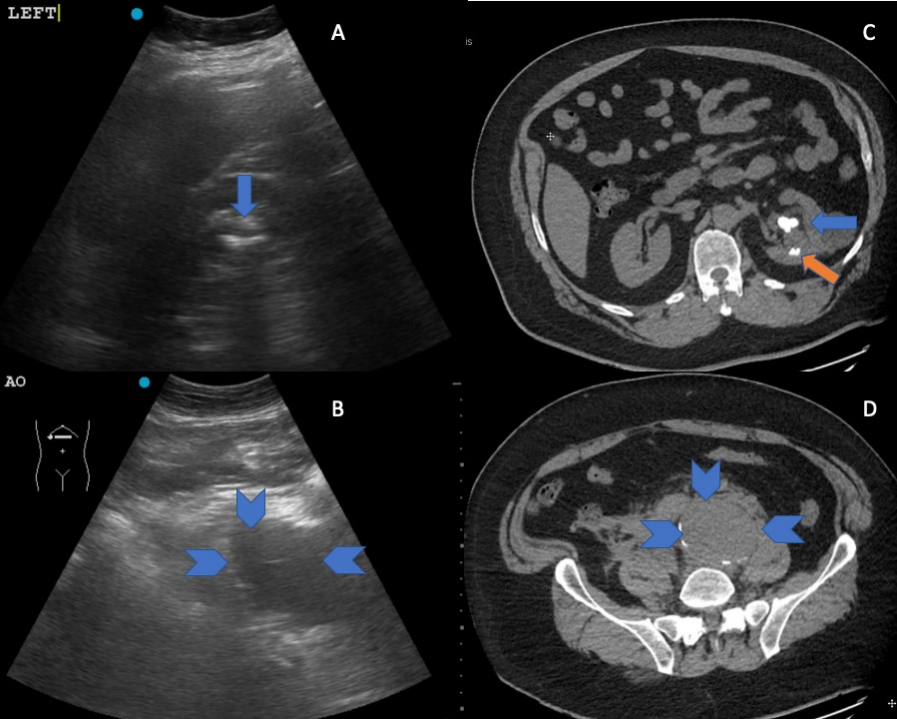
A) POCUS image of left kidney with large stone in the renal pelvis (blue arrow) and hydronephrosis. B) left iliac artery (blue chevrons). C) CT image of left kidney the renal pelvis stone (blue arrow) and lower pole non-obstructing stones (orange arrow) and D) left iliac artery with aneurysm (blue chevrons).

Point of care ultrasound (POCUS) was performed, with targeted views of the kidneys and bladder given the patient’s history of nephrolithiasis, as well as complete visualization of the aorta to the iliac bifurcation given the patient’s presentation with undifferentiated flank pain in accordance with emergency medicine POCUS recommendations 1, 2, 3. The ultrasound revealing a large left renal stone with associated hydronephrosis (Figure 1A), a non-aneurysmal aorta, and an enlarged left iliac artery (Figure 1B, online Video S1). The presence of the dilated left iliac artery prompted immediate consultative imaging rather than the common diagnostic pathway of next day outpatient consultative imaging. A CT scan confirmed the presence of multiple left-sided renal stones with hydronephrosis including a 2cm cluster in the renal pelvis (Figure 1C) and a 6.7cm ruptured left isolated iliac artery aneurysm (IAA; Figure 1D). 

IAA typically presents in conjunction with abdominal aortic aneurysm 4, commonly mimicking renal colic 5, and can present with hydronephrosis without renal stones 6. Isolated IAA is a rare diagnosis accounting for approximately 0.4% to 1.9% of all arterial aneurysms 7. Isolated IAA are at a particular high risk for rupture 6. Rupture of an isolated IAA carries a mortality risk of 50-75% 4, 6. 

IAA is an uncommon, but important diagnosis that might mimick other more frequently encountered disease processes. IAA should be considered for patients with histories incongruous with their physical exam findings. Abdominal pain, dysuria, urinary frequency or urgency, constipation, hydronephrosis, pelvic masses are common historical features and findings for IAA 4. Most IAAs are discovered incidentally on imaging ordered for other indications 7. The aorta and renal studies are typically performed together as both renal colic and symptomatic ruptured AAA are relatively common causes of severe acute flank and abdominal pain 1, 2, 3. POCUS is typically performed in a targeted manner to the patient’s symptoms in contrast to comprehensive consultative radiology studies. In our patient there was a real possibility that his pain was caused by a ruptured AAA, or in this case an IAA as well as renal colic.

This case highlights the importance of systematically performing related POCUS studies. Here, the operator systematically searched for AAA in spite of already having identified urolithiasis and hydronephrosis correlating with the patient’s symptoms. The systematic use of POCUS, prevented the pitfall of anchoring bias and premature closure bias, both recognized as common sources of bias in diagnosis 8. AAA is a life-threatening diagnosis, with a varied presentation, of which physical exam is particularly unreliable in detection 9. 

As POCUS presents a rapid, sensitive, and accurate assessment tool for examination of the abdominal aorta, particularly in the symptomatic population 3, it is a useful test for avoiding premature closure in patients with undifferentiated severe flank or abdominal pain, particularly in patients >50 years old who have a higher risk of aneurysm compared to younger populations 2, 3. 

The patient was transferred to the operating room and underwent an uncomplicated endovascular repair. He was discharged home on post-operative day 2. Of note, he also underwent laser lithotripsy and basket retrieval of nephrolithiasis. The use of POCUS in this patient facilitated the diagnoses of dual conditions warranting subspecialty intervention.

## Statement of Patient Consent

The authors certify that informed consent was obtained from the patient. The patient has consented to the use of images, video clips, and information regarding his condition and treatment to be published within the journal.

## Disclosures

The authors have no conflicts of interest to declare. 

## Supplementary Material

 Video S1 First clip: left kidney with large stone in the renal pelvis and hydronephrosis. Second clip: left iliac artery.
